# Correction of RNA-Binding Protein CUGBP1 and GSK3β Signaling as Therapeutic Approach for Congenital and Adult Myotonic Dystrophy Type 1

**DOI:** 10.3390/ijms21010094

**Published:** 2019-12-21

**Authors:** Lubov Timchenko

**Affiliations:** Departments of Neurology and Pediatrics, Cincinnati Children’s Hospital Medical Center and the University of Cincinnati, Cincinnati, OH 45229, USA; Lubov.Timchenko@cchmc.org; Tel.: +1-513-803-0768

**Keywords:** myotonic dystrophy, congenital myotonic dystrophy, CUGBP1, GSK3β signaling, GSK3 inhibitors

## Abstract

Myotonic dystrophy type 1 (DM1) is a complex genetic disease affecting many tissues. DM1 is caused by an expansion of CTG repeats in the 3′-UTR of the *DMPK* gene. The mechanistic studies of DM1 suggested that *DMPK* mRNA, containing expanded CUG repeats, is a major therapeutic target in DM1. Therefore, the removal of the toxic RNA became a primary focus of the therapeutic development in DM1 during the last decade. However, a cure for this devastating disease has not been found. Whereas the degradation of toxic RNA remains a preferential approach for the reduction of DM1 pathology, other approaches targeting early toxic events downstream of the mutant RNA could be also considered. In this review, we discuss the beneficial role of the restoring of the RNA-binding protein, CUGBP1/CELF1, in the correction of DM1 pathology. It has been recently found that the normalization of CUGBP1 activity with the inhibitors of GSK3 has a positive effect on the reduction of skeletal muscle and CNS pathologies in DM1 mouse models. Surprisingly, the inhibitor of GSK3, tideglusib also reduced the toxic CUG-containing RNA. Thus, the development of the therapeutics, based on the correction of the GSK3β-CUGBP1 pathway, is a promising option for this complex disease.

## 1. Introduction: Complex Molecular Pathophysiology of DM1

Myotonic dystrophy type 1 (DM1) is a complex genetic disease affecting many tissues, including skeletal and cardiac muscles, the brain, the eye and the endocrine system [1]. DM1 is characterized by a strong variability of phenotype. Some patients with DM1 might be asymptomatic, whereas other patients might have mild disease with progression of symptoms with age. Most severe form of DM1, congenital DM1 (CDM1), affects children at birth or after birth. CDM1 is associated with a neuro-motor deficit, severe muscle weakness and cognitive defects.

DM1 is caused by an expanded track of CTG repeats of various lengths in the 3′-untranslated region (UTR) of the myotonin protein kinase (*DMPK*) gene on the chromosome 19q [2]. Numerous molecular studies showed that DM1 is a “RNA disease”, which is caused by the accumulation of RNA CUG repeats that misregulate RNA metabolism in patients’ tissues via specific RNA-binding proteins (rev in [3,4]). Whereas majority of the studies in DM1 research were focused on two RNA-binding proteins, CUGBP1 and MBNL1, it appears that other RNA-binding proteins also contribute to DM1 pathogenesis. In addition to CUGBP and MBNL families, the growing list of RNA-binding proteins, affected in DM1, includes RNA helicases DDX5 [5,6] and DDX6 [7], Staufen [8,9,10,11] and hnRNP H [12].

The complexity of DM1 pathogenesis seems to be even higher and includes recently identified repeat associated non-ATG (RAN) translation, driven by the non-coding CUG repeats [13]. Thus, the definition of the 3′-uncoding region of the mutant *DMPK* mRNA might be revised because this region of the mutant mRNA might encode short peptides in all open-reading frames. Although the role of these peptides in DM1 pathogenesis remains to be investigated, it is likely that they contribute to DM1 pathology.

The identification of several early mechanistic pathways, initiated by the mutant CUG repeats in DM1 cells, revealed potential therapeutic targets for DM1, besides the toxic RNA. In this review, we will focus on the role of CUG repeats in the misregulation of the GSK3β-CUGBP1 pathway in DM1 pathogenesis and discuss recent findings, which describe how correction of this pathway may reduce DM1 and CDM1 pathology.

## 2. Why Are Expanded RNA CUG Repeats Toxic?

After the discovery of the DM1 mutation (expansion of CTG repeats), the main question in DM1 studies was related to the mechanisms by which the expanded CTG repeats in the 3′-UTR of the mutant RNA cause the disease. First attempts to identify the mechanism of DM1 were focused on the role of DMPK protein kinase or genes surrounding *DMPK* (rev in [14]). These studies suggested that although DMPK kinase and proteins, encoded by the genes in the *DMPK* locus, might be involved in DM1 pathophysiology, they likely represent only a portion of very complex DM1 pathogenesis. It took a long time to determine that the expanded CTG repeats harm cell functions via RNA CUG repeats.

The breakthrough in the understanding of the DM1 mechanism was inspired by the pioneering work from Dr. Blau’s group, which investigated the regulation of muscle differentiation. This group surprisingly found that the 3′-UTRs of the muscle genes might contain regulatory elements that play a critical role in the control of muscle growth and differentiation [15]. In addition, other studies suggested that the mutant *DMPK* mRNA in DM1 cells might have a trans-dominant effect on RNA metabolism, altering accumulation of poly(A)^+^ RNA in DM1 [16,17]. These studies, together with relatively mild phenotype in *Dmpk* knock out mouse models [18,19], created a background for an entirely new hypothesis for the role of the 3′-UTR of the mutant *DMPK* mRNA in the disease pathogenesis. This hypothesis suggested that the mutant 3′-UTR of *DMPK* mRNA might have a pathologic effect independently of the 5′ regulatory region of the mutant *DMPK* mRNA or DMPK protein dysfunction [20,21,22].

Initially, toxic effects of the mutant 3′-UTR of *DMPK*, containing expanded CUG repeats, were investigated in several directions, including (a) examination of the intracellular properties of the mutant *DMPK* mRNA within DM1 cells; (b) identification of RNA-binding proteins, interacting with CUG repeats and (c) examination of the role of the mutant 3′-UTR of the *DMPK* mRNA in normal myogenesis in muscle cells and in mouse models.

In 1995, Dr. Singer’s group tested the hypothesis whether the mutant *DMPK* mRNA is blocked in the nuclei, preventing its transport from the nuclei to cytoplasm, causing a reduction of *DMPK* translation. In the course of these studies, they found that the mutant *DMPK* mRNA is detected in both nuclei and in the cytoplasm in DM1 fibroblasts; however, the appearance of the mutant transcripts in cytoplasm and nuclei was different. Whereas cytoplasmic mutant *DMPK* mRNA was dispersed, nuclear CUG-containing transcripts were observed in a form of complexes or foci [23]. Other studies suggested that the mutant *DMPK* mRNA is completely blocked in the nuclei [24]. Whereas more investigations are needed to determine how CUG foci are formed and whether they are toxic or protective, the presence of CUG foci in DM1 cells clearly demonstrates the abnormal accumulation of the mutant *DMPK* mRNA in DM1. This finding provided not only a simple assay for the detection of the mutant CUG-containing transcripts in DM1 cells, but also created an opportunity to use fluorescence “in situ” hybridization (FISH) assay for testing drugs that might remove the toxic mRNA in DM1. The identification of CUG-containing aggregates also opened a new direction in search for RNA-binding proteins that interact with the expanded CUG repeats.

First CUG RNA-binding protein, CUGBP1 (also known as CUG-BP, NAB50 or CELF1) was identified during studies, addressing the hypothesis if the mutant CUG repeats display their pathologic function through binding to some RNA-binding proteins, sequestering them away from normal functions [20,21,22]. CUGBP1 was identified based on its strong affinity to synthetic RNA, containing short CUG repeats (CUG_8_) [20,21]. Later, it was shown that CUGBP1 belongs to a family of highly homologous RNA-binding proteins. Second member of this family, CUGBP2 (also known as ETR3, NAPOR, BRUNO3 or CELF2), was also described [25]. CUGBP1 and ETR3 like the family of proteins (CELF) belongs to a large super family of ELAV (embryonic lethal abnormal visual system) RNA-binding proteins that includes highly conserve RNA-binding proteins, which play a critical role in cell development, growth and differentiation especially in the CNS system [26].

Although initially CUGBP1 was identified as a CUG-binding protein with altered RNA-binding activity in DM1, it was questioned whether CUGBP1 binds to long CUG repeats or whether it binds to CUG repeats at all. Some studies showed that CUGBP1 specifically interacts with normal *DMPK* mRNA in vitro [21]. CUGBP1 also interacts with the mutant *DMPK* mRNA in cellular extracts from human heart specimens from patients with DM1 [27]. Biochemical studies indicated that in human hearts from DM1 patients, CUGBP1 is detected in high molecular weight RNA-protein complexes, containing CUG repeats; whereas in human hearts from control (not affected by DM1) patients, CUGBP1 exists as a free protein [27]. FISH analysis, however, did not detect CUGBP1 signals in the complexes with CUG foci in DM1 cells [28,29].

Four years after discovery of CUGBP1, Dr. Swanson’s group used *DMPK* mRNA, containing approximately 100 CUG repeats as a bait to identify additional CUG-binding proteins that specifically interact with long CUG repeats. This study led to the identification of the first member of MBNL family of proteins, MBNL1, which binds to the nuclear CUG foci in DM1 cells [28]. The binding of MBNL1 to the mutant CUG aggregates does not affect MBNL1 protein levels but reduces its activity.

In contract to MBNL1, CUGBP1 does not bind to CUG foci [28]. The lack of the visual binding of CUGBP1 to CUG foci in the FISH assay might be due to the binding of CUGBP1 to the mutant *DMPK* mRNA outside of CUG foci. As shown by Dr. Singer’s group, the mutant *DMPK* mRNA in DM1 cells is not restricted to CUG aggregates or foci and it is dispersed throughout the cytoplasm [23]. In agreement, CUGBP1 was initially identified by the electrophoretic mobility shift assay (EMSA), using ^32^P-labeled CUG_8_ RNA from cytoplasmic Hela proteins [20,21]. It is also possible that some portion of the mutant *DMPK* mRNA exists in soluble form and CUGBP1 binds to soluble CUG repeats. Such binding is easily detected by the biochemical methods [27] but might not be detected by a FISH assay. It is also possible that the binding of CUGBP1 to the mutant CUG foci is weak. The later suggestion agrees with the findings of electron microscopy analysis, which showed that CUGBP1 binds to CUG repeats at the base of the double stranded CUG hairpins, whereas MBNL1 interacts with the dsCUG hairpins along the stem [29].

Investigations of CUGBP1 functions in human DM1 cells and in DM1 tissue specimens (skeletal and cardiac muscles) showed that the mutant CUG repeats have a complex effect on CUGBP1. First, CUGBP1 RNA-binding activity is altered in DM1 cells [30]. Second, the protein levels of total CUGBP1 are increased in DM1. Comparison of CUGBP1 levels in normal and DM1 heart specimens showed that CUGBP1 is elevated in DM1 hearts [27]. CUGBP1 levels are also increased in human muscle biopsies from patients with DM1 [31].

The increase of CUGBP1 in DM1 patients occurs at the protein level due to stabilization of CUGBP1 within complexes, containing the mutant *DMPK* mRNA [27]. Dr. Cooper’s group showed that the increase of CUGBP1 stability in DM1 is mediated by the increased phosphorylation of CUGBP1 by PKC kinase [32]. Together, these findings suggest that PKC-mediated phosphorylation might control the affinity of CUGBP1 to the mutant *DMPK* mRNA (mainly in soluble form and/or at the base of dsCUG hairpins), stabilizing CUGBP1 and increasing its levels.

Thus, the expanded CUG repeats are toxic because they accumulate in DM1 cells, affecting RNA-binding proteins, such as CUGBP1 and MBNL1, changing their expression and activity. Figure 1 summarizes the studies, describing the effects of CUG repeats on CUGBP1 and MBNL1.

Deregulation of CUGBP1 and MBNL1, caused by the mutant CUG repeats, leads to the abnormal RNA processing in DM1. The toxicity of CUG repeats was confirmed in DM1 cell and mouse models, in which pure CUG repeats with or without the surrounding sequences from the 3′-UTR of the mutant *DMPK* mRNA cause a delay of myogenesis and lead to muscle pathology [33,34,35,36].

## 3. The Misregulation of CUGBP1 in DM1 Leads to the Abnormal RNA Processing at Multiple Levels

### 3.1. The Role of Phosphorylation in the Regulation of CUGBP1 Intracellular Localization and Translational Functions of CUGBP1

CUGBP1 is a multifunctional protein that regulates RNA processing at the levels of translation, splicing and RNA stability. The increase of the total levels of CUGBP1 in DM1 suggests that the activity of CUGBP1 is also increased in DM1. However, it was shown, that translational function of CUGBP1 depends on post-translational modifications and that CUGBP1 might exist as two isoforms: as an activator of translation and as a repressor of translation (rev in [3]). The activity of these isoforms is regulated by phosphorylation. As the result, in addition to the negative effect of the increased protein levels of CUGBP1 on DM1 pathology, abnormal phosphorylation of CUGBP1 plays a critical role in DM1 pathogenesis.

Besides CUGBP1 activity, phosphorylation regulates CUGBP1 intra-cellular localization. Two critical sites of phosphorylation have been identified in CUGBP1: Ser-28 and Ser-302.

Phosphorylation of CUGBP1 at Ser-28 by AKT kinase is important for cytoplasmic localization of CUGBP1 [37]. Phosphorylation of CUGBP1 at specific sites might also control the affinity of CUGBP1 to certain mRNA targets. As shown, the phosphorylation of CUGBP1 by AKT increases its binding to cyclin D1 mRNA, affecting proliferation of human myoblasts [38]. This phosphorylation site is also important for CUGBP1 function in degradation of mRNAs in cancer cells. As the result, un-phosphorylated and phosphorylated at Ser-28 CUGBP1 might bind to different sets of mRNAs in normal and cancer cells causing a malignant phenotype [39].

Translational function of CUGBP1 is controlled by phosphorylation of CUGBP1 at Ser-302 by cyclin D3-CDK4 kinase (Figure 2). The translational function of CUGBP1 is connected to many cellular processes, including control of cell proliferation and differentiation.

Phospho-Ser-302-CUGBP1 interacts with the active form of eIF2α and protein–protein complexes of phospho-Ser-302-CUGBP1 and active eIF2α promote translation of mRNAs on polysomes [40,41]. For instance, during myoblasts differentiation, CUGBP1, phosphorylated by the cyclin D3-CDK4/6 at Ser-302, binds stronger to mRNAs, encoding transcription factor C/EBPβ and cyclin-dependent kinase inhibitor, p21, important for cell differentiation [38,42]. Respectively, the protein levels of CUGBP1 translational targets, C/EBPβ and p21 are increased in normal myotubes [38,42]. CUGBP1 regulates translation of these mRNAs via binding to Guanine Cytosine (GC)-rich regions within the 5′-UTRs of mRNA targets [42,43]. CUGBP1 also upregulates translation of myocyte enhancer factor MEF2A via binding to GC-rich site in *MEF2A* mRNA [44].

Comparison of CUGBP1 phosphorylation at Ser-302 in normal and DM1 muscle cells showed that phosphorylation of CUGBP1 at Ser-302 is reduced in DM1 due to a reduction of cyclin D3 [38], which binds to and activates proliferation-linked cyclin D-dependent kinases 4 and 6 [45]. Cyclin D3 reduction in DM1 myotubes was confirmed by the analysis of cyclin D3 in human skeletal muscle biopsies from patients with DM1 [31,38].

It has been shown that the protein levels of cyclin D3 are regulated at the level of stability via two pathways: (a) by interaction of cyclin D3 with retinoblastoma protein (Rb) and by (b) phosphorylation of cyclin D3 by GSK3β kinase. Cyclin D3, bound to Rb, is stabilized; whereas cyclin D3, phosphorylated by GSK3β is subjected to degradation by the Ub-proteasome system [46,47]. Examination of the interactions of cyclin D3 with Rb in skeletal muscle biopsies from patients with DM1 showed that the amounts of cyclin D3-Rb complexes are not altered in DM1 [31]. However, GSK3β, which phosphorylates cyclin D3, causing its reduction, was elevated in DM1 muscle biopsies [31]. Analysis of DM1 mouse models, *HSA^LR^* mice that express 250 CTG repeats in the 3′-UTR of human skeletal muscle actin [34] and DMSXL mice, which carry the human *DMPK* gene with more than 1,000 CTG repeats in the 3′-UTR [48] showed that GSK3β is increased and cyclin D3 is respectively reduced in skeletal muscle of these mice [31,49,50]. GSK3β is also abnormally elevated in brains of DMSXL mice [50].

It is known that GSK3β exists as two isoforms, active and inactive GSK3β [51]. Active GSK3β is phosphorylated at Tyr-216; whereas inactive GSK3β is phosphorylated at Ser-9. Immunoanalysis of GSK3β isoforms in DM1 showed that active GSK3β is elevated in human muscle biopsies from patients with classic form of DM1 [31]. Thus, in DM1, both total levels of GSK3β and the levels of active, phosphorylated at Tyr-216, GSK3β are increased.

The mechanism of the increase of GSK3β in DM1 remains to be investigated. One of the possible mechanisms suggests that the mutant CUG repeats increase stability of GSK3β due to elevation of phosphorylation of GSK3β at Tyr-216 [31]. The increase of GSK3β stability, accompanied by the elevation of GSK3β phosphorylation at Tyr-216 is caused by the mutant CUG repeats because GSK3β is elevated in skeletal muscle of *HSA^LR^* mice, expressing 250 pure CUG repeats [31]. However, the pathway by which the mutant CUG repeats promote phosphorylation of GSK3β at Tyr-216 remains to be investigated. It has been shown that GSK3β can phosphorylate itself at Tyr-216 due to autophosphorylation, increasing its own activity [52].

Another possible mechanism of the pathological elevation of active GSK3β in DM1 might be associated with the activation of protein kinase R (PKR) [37,53]. It was shown that CUG repeats cause cellular stress increasing PKR, activated by double-stranded RNA. PKR kinase might be involved in the signaling pathway, phosphorylating GSK3β at Tyr-216 in DM1. Although PKR kinase is not tyrosine-specific kinase, it can activate tyrosine specific Fyn kinase [54]. In addition, Ca^2+^-dependent tyrosine kinase Pyk2 might phosphorylate GSK3β at Tyr-216 in DM1, because Pyk2 activates GSK3β in vitro and in neuronal cells during lysophosphatidic acid (LPA)-induced neurite retraction [55,56].

### 3.2. The Role of Splicing Activity of CUGBP1 in DM1

Alterations of CUGBP1 levels and its activity have a critical effect on RNA metabolism in DM1 tissues. It has been shown that CUGBP1 has numerous RNA targets and can regulate mRNA processing in both nuclei and cytoplasm [30]. In the nuclei, CUGBP1 regulates splicing. Identification of altered splicing of mRNA, encoding cardiac Troponin T (cTnT) in DM1, regulated by CUGBP1, opened a new direction in DM1, focused on the role of the global missplicing in DM1 pathogenesis [57]. The significance of CUGBP1-mediated missplicing of *cTnT* in DM1 was demonstrated by the experiments in which over-expression of CUGBP1 in normal human myoblasts caused the same abnormal pattern of *cTnT* splicing as forced expression of CUG repeats [57].

After identification of *cTnT* as a splicing target of CUGBP1, numerous splicing targets of CUGBP1 have been identified in different tissues, including skeletal and cardiac muscles [36,58,59,60,61,62]. The list of the splicing targets, regulated by CUGBP1, includes mRNAs encoding proteins involved in various important cellular functions. For instance, one of the CUGBP1 splicing targets, Ankyrin 2 (Ank-2) is associated with linking membrane proteins to the cytoskeleton [36]. Another splicing target of CUGBP1 encodes a Fragile X mental retardation-related protein (Fxr1), which is involved in mRNA transport [36]. Other splicing targets of CUGBP1 include mRNAs, encoding F actin capping protein β subunit (Capzb), which contributes to filament growth [36]; muscle-specific chloride ion channel 1, that is needed for electrical stability of the membranes in skeletal muscle, contributing to myotonia in DM1 [59]; and insulin receptor, which plays a key role in the glucose homeostasis [60]. Some splicing targets CUGBP1 might bind directly, whereas splicing of other mRNAs CUGBP1 may regulate in cooperation with other RNA-binding proteins [62].

A recent report suggests that the phosphorylation of CUGBP1 at Ser-302 by the cyclin D3-CDK4/6 might affect splicing activity of CUGBP1. It was shown that CUGBP1 is increased in human patients and in mouse models with neurogenic muscle atrophy [63]. The upregulation of CUGBP1 in neurogenic muscle atrophy directly affects the alternative splicing of ryanodine receptor 1 (RyR1), a major receptor in skeletal muscle, regulating Ca2^+^ homeostasis, altering calcium release in myofibers. Since the levels of cyclin D3 and CDK4 are increased in the neurogenic muscle atrophy [63], it is possible that the phosphorylation of CUGBP1 at Ser-302 might affect splicing activity of CUGBP1 towards RyR1 in neurogenic atrophy as well as in DM1 muscle pathology.

### 3.3. CUGBP1 Is a Regulator of mRNA Stability

Recent studies designated CUGBP1 as a major protein factor, regulating mRNA stability. It has been shown that CUGBP1 binds to the AU-rich elements (ARE) or sequences flanking the AREs in the 3′-UTRs of a tumor necrosis factor α (TNFα) and c-fos mRNAs [64]. CUGBP1 also possesses a polyadenylation activity [64,65,66]. Once CUGBP1 binds to mRNAs, it recruits the poly(A) specific ribonuclease PARN, promoting deadenylation of its mRNA targets [64].

Early studies identified the GU repeat as a binding motif for CUGBP1 [67,68,69,70]. Thorough examination of CUGBP1 targets in human cells showed that CUGBP1 binds to the GU-rich element (GRE), located in the 3′-UTRs of short-lived mRNAs, important for cell proliferation, cell growth, motility and cell survival [71,72,73,74,75]. Since CUGBP1 binds to hundreds of mRNAs, stability of which might be regulated by CUGBP1, it has been suggested that CUGBP1 and GREs comprise a specific posttranscriptional regulatory network in human cells that might turn on and off specific regulators of the cell cycle and apoptosis at the level of mRNA degradation [71]. Regulation of degradation of mRNAs, targets of CUGBP1, is closely linked to the translational function of CUGBP1. Phosphorylation of CUGBP1 might contribute to the regulation of the processing of GRE-containing transcripts, contributing to the uncontrolled proliferation in cancer cells [39]. Similar pathways might function in DM1 cells. Examination of CUGBP1 mRNA targets in C2C12 myoblasts showed that CUGBP1 binds to hundreds of mRNAs via ARE or GRE elements [74]. One of the CUGBP1 targets in C2C12 myoblasts is mRNA, encoding a myogenic transcription factor, MyoD. CUGBP1 might regulate *MyoD* stability in coordination with RNA-binding protein, a human antigen R (HuR) [74].

The CUGBP1-dependent regulation of mRNA stability in DM1 skeletal and cardiac muscles and in the DM1 brain remains to be investigated. It is expected that CUGBP1 might deregulate large number of the GRE-containing mRNAs in DM1. This expectation is based on a large number of mammalian mRNAs with the GRE elements and on the knowledge that CUGBP1 is a major GRE-binding protein. In agreement, hundreds of mRNAs, binding to CUGBP1 via their 3′-UTRs have been identified in mouse skeletal muscle and in the heart [62,76].

The number of CUGBP1 targets in DM1 and CDM1 could be even greater because CUGBP1 functions in cooperation with other RNA-proteins and microRNAs in the regulation of RNA processing. For instance, CUGBP1 controls cell proliferation by cooperating with HuR via regulation of translation of *MYC* and *CDK4* mRNAs [77,78]. The mechanism of the regulation of CDK4 translation includes the binding of CUGBP1 to GRE and recruitment of *CDK4* mRNA (in cooperation with microRNA-222) to the processing bodies (PBs), which contain translational repressors, RNA-binding proteins, TIAR and TIA. This causes *CDK4* decay and repression of *CDK4* translation [78]. This mechanism of deregulation of translation might be involved in DM1 pathogenesis because CUGBP1 accumulates in DM1 myoblasts in stress granules, containing TIA protein [37].

Other examples of the cooperation of CUGBP1 with HuR include possible effect of CUGBP1 on inflammation via regulation of translation of occludin, which controls the integrity of tight junctions (TJ), important for cell polarity and control of diffusion of allergens, toxins and pathogens [79]. In this pathway, HuR displaces CUGBP1 from occludin mRNA, preventing its translocation to the PBs and its degradation. CUGBP1 also cooperates with HuR in the regulation of translation of a protein of the extracellular matrix (ECM), E-cadherin [80].

CUGBP1 is expressed in many types of cells, where it regulates tissue-specific mRNAs. Therefore, alteration of CUGBP1 protein levels and its activity in DM1, caused by the mutant CUG repeats, might misregulate CUGBP1 targets in all affected tissues, including skeletal and cardiac muscles and brain. Numerous mRNAs are regulated by CUGBP1 in the heart. This list of mRNAs includes mRNAs encoding connexin 43 (Cx43), a protein abundant in cardiac gap junction and antioxidant enzyme, heme oxygenase 1 (HO-1) [81,82]. CUGBP1 regulates stability of *Cx43* mRNA via the binding to the UG element in the 3′-UTR of *Cx43*, causing its degradation. This target of CUGBP1 might play a role in cardiac dysfunction in DM1. CUGBP1 also binds to *HO-1* mRNA, inhibiting its stability [82]. Upregulation of this protein, controlled by CUGBP1, might prevent cardiac hypertrophy.

Important, that the list of CUGBP1 mRNA targets in human cells, includes mRNAs, encoding RNA-binding proteins, such as MBNL1, hnRNP-A3, hnRNP-K, hnRNP-D, hnRNP-DL, hnRNP-R, hnRNP-A1, hnRNP-U and others [83]. Therefore, CUGBP1 was described as “a master regulator” of RNA processing that might regulate other RNA-binding proteins, which in turn, regulate their own sets of mRNAs [83].

Microarray analysis of genes misregulated in mouse tissues with deleted CUGBP1 (*Celf1* knock out mice) supports the role of CUGBP1 as “a master regulator” of RNA processing. Several brain mRNAs, encoding RNA-binding proteins, such as Mbnl3, Rbm45 and Smn1 were identified downstream of CUGBP1 [49,50]. RNA-binding protein, RBM45 (RNA binding motif 45), is involved in cell development [84]. SMN1 (spinal motor neuron 1) is associated with the maintenance of the motor neurons [85]. MBNL3 belongs to a family of MBNL proteins that are affected in DM [86,87,88]. Misregulation of CUGBP1 in DM1 might affect these mRNAs (directly or indirectly); therefore, the number of genes, disrupted by CUGBP1 in DM1, might be great because each of these RNA-binding proteins controls own sets of mRNAs.

Gene pathway analysis in mouse tissues from *Celf1* knock out mice showed that CUGBP1 functions in skeletal muscle and in brain are linked to development, nucleotide metabolism, receptor signaling, cell important and export, protein folding, cell differentiation and protein turnover [49,50]. In agreement with the deregulation of CUGBP1 targets, CUGBP1 function in vivo is linked to development, growth and myogenesis [44,89,90,91,92,93]. Overexpression of CUGBP1 in skeletal muscle leads to muscle wasting; whereas the increase of CUGBP1 in the heart causes dilated cardiomyopathy [90,91]. Overexpression of CUGBP1 lacking the nuclear localization signal also affects skeletal muscle [93]. It remains to determine the role of CUGBP1 dysfunction in other tissues, affected in DM1. It appears that high or low levels of CUGBP1 are equally toxic for myogenesis because skeletal muscle is affected in mice with deleted or over-expressed CUGBP1 [44,49,89]. Current data also suggest that, in addition to the importance of the proper levels of CUGBP1 for normal cell function, the status of CUGBP1 phosphorylation plays an essential role. Therefore, based on its function, CUGBP1 has potential to disrupt multiple molecular and cellular events in DM1 tissues during lifespan.

## 4. Inhibitors of GSK3 in DM1 and CDM1 Therapeutic Approaches

How to correct CUGBP1 activity in CDM1 and DM1? Since PKC phosphorylation contributes to the stabilization of CUGBP1, the inhibitors of PKC could normalize CUGBP1 levels in DM1 [94]. In support, one of the kinase inhibitors, identified by the screening of the panel of kinase inhibitors according to their ability to reduce the mutant CUG aggregates, also normalized CUGBP1 protein levels [95].

To restore CUGBP1 activity in DM1 cell and mouse models, various inhibitors of GSK3 have been applied. Treatments of adult mice with a classic form of DM1 (*HSA^LR^* model) with lithium for two weeks reduced myopathy, myotonia and the grip weakness (Table 1) [31]. The improvement of muscle function in these mice was accompanied by the normalization of GSK3β and correction of its substrate cyclin D3. CUGBP1 activity was also recovered because CUGBP1 restored its binding to the active eIF2α in skeletal muscle of the treated mice [31].

The positive effect of the normalization of GSK3β on DM1 phenotype in adult *HSA^LR^* muscle, treated with lithium, was reproduced by the treatment of these mice with a non-ATP competitive inhibitor of GSK3, TDZD-8 (4-benzyl-2-methyl-1,2,4-thiadiazolidine-3,5-dione or NP 01139) [31].TDZD-8 increased the number of activated myogenic satellite cells in the skeletal muscle of the treated *HSA^LR^* mice, accompanied by the increase of a marker of myogenic satellite cells, transcription factor Pax-7 [31]. This finding suggests that the inhibitors of GSK3 might activate muscle regeneration in DM1 mouse model.

Another small molecule inhibitor of GSK3, indirubin and its homologue with increased solubility, BIO (6-bromoindirubin-3-oxime), also reduced myopathy and the grip weakness in adult *HSA^LR^* mice [49]. Important, that BIO, shortly applied in young *HSA^LR^* mice prior the development of the overt DM1 muscle phenotype, almost prevented the development of muscle pathology for 9.5 months [49]. Prevention of DM1 muscle pathology in adult *HSA^LR^* mice, shortly treated with BIO at a young age, was accompanied by the correction of GSK3β and cyclin D3, normalization of CUGBP1 levels and its activity, determined by the interaction of CUGBP1 with active eIF2α [49].

Like TDZD-8, BIO also increased Pax-7 in skeletal muscle of adult *HSA^LR^* mice, treated at a young age, suggesting that the prevention of muscle pathology in the treated mice might be associated with the activation of muscle regeneration at a young age prior the development of DM1 muscle pathology [49]. It appears that these treatments have a long beneficial effect on *HSA^LR^* muscle without obvious negative effects on mice. The mechanism of the prevention of muscle pathology in *HSA^LR^* mice treated with BIO at a young age is associated with normalization of CUGBP1 activity in young *HSA^LR^* muscle, which might lead to the correction of the expression of the myogenic targets, downstream of CUGBP1, such as Rbm45; a transcription regulator Lef1; a protein, regulating myoblasts migration and synaptogenesis, doublecortin (Dcx) and others [49]. It is also important that the correction of CUGBP1 with the inhibitors of GSK3 might improve the extracellular matrix (ECM) and cell adhesion via correction of the downstream CUGBP1 targets, such as a collagen 4A (Col 4A), associated with the basement membrane and collagen 13 (Col13), that plays a role in the neuromuscular junction [49]. It has been suggested that the improvement of ECM in young *HSA^LR^* mice, treated with BIO at a young age, might lead to the improvement of myoblasts migration, supporting the maintenance of myogenesis in *HSA^LR^* mice overtime [49]. The myogenic downstream targets of CUGBP1, RBM45, LEF1, DCX and Col4A are truly important for DM1 skeletal muscle pathology because these proteins are misregulated in skeletal muscle biopsies from patients with pediatric CDM1 [49].

These findings created a background for the testing of the inhibitors of GSK3 in the pre-clinical and clinical studies for DM1 and CDM1. A clinical trial-ready small molecule inhibitor of GSK3, tideglusib (TG), was used in the pre-clinical studies for DM1 and CDM1 [50] and in a Phase IIa clinical trial for adolescent and adult patients with DM1 [96].

Studies in DM1 mouse models showed that TG treatments are beneficial for the reduction of myopathy and a recovery of the grip strength in adult *HSA^LR^* mice [50]. As found [50], a positive effect of TG was dose-dependent without obvious deleterious effects on Wild Type (WT) or adult *HSA^LR^* mice. The improvement of skeletal muscle in the TG-treated mice was accompanied by the normalization of GSK3β, and normalization of CUGBP1 downstream myogenic targets such as Dcx and Rbm45. Surprisingly, the TG treatments were also beneficial for the reduction of the mutant CUG-containing RNA and for the decrease of CUG foci in skeletal muscle of adult *HSA^LR^* mice [50]. The reduction of CUG repeats in the TG-treated adult *HSA^LR^* mice led to the improvement of missplicing of some tested mRNAs, such as *Serca1* and *Cypher* [50].

TG also had a positive effect in a mouse model for DM1 (DMSXL mice) [50], which express a human *DMPK* gene with long (more than 1000) CTG repeats in the range, observed in severely affected patients with CDM1 [48]. TG reduced muscle histopathology in adult DMSXL mice, normalizing GSK3β and one of the GSK3β substrates, cyclin D3 (Ref. [50], Table 2). As in the adult *HSA^LR^* mice, treated with other inhibitors of GSK3 TDZD-8 or BIO), TG also increased Pax-7 levels in the skeletal muscle of adult DMSXL mice [50].

In contrast to *HSA^LR^* mice, which express pure CUG repeats, driven by human skeletal muscle actin promoter mainly in skeletal muscle [34], DMSXL mice express human *DMPK* mRNA with long CUG repeats under *DMPK* promoter in all tissues, where DMPK functions [48]. Due to this, the DMSXL model allows examination of the GSK3β-CUGBP1 pathway in CNS. This knowledge is important to determine if the inhibitors of GSK3 could be used to correct CNS defects, associated with DM1 and CDM1. Examination of GSK3β in the brains of DMSXL mice showed that GSK3β is abnormally elevated in the DMSXL brains and that TG treatments normalized GSK3β in the brains of DMSXL mice, restoring CUGBP1 activity [50].

It is known that long CTG repeat expansions cause CDM1, affecting patients before or after birth [1]. Homozygous DMSXL mice with long CTG repeat expansions are characterized by the increased postnatal mortality and delayed survivors’ growth with increased anxiety [48,97]. Important, that the prenatal correction of GSK3β in DMSXL mice with TG significantly increased the survival rate of homozygous DMSXL mice (females) [50]. In addition, prenatally TG-treated DMSXL mice, grew faster, were stronger and did not develop anxiety at a young age [50]. Improvement of the neuromotor activity in DMSXL mice, generated by the TG-treated females, was accompanied by the correction of the downstream targets of CUGBP1 in the brain, such as *Rbm45*, *Smn1*, *Mbnl3* and *Fgf-2* [50]. These findings demonstrate the potential of the prenatal treatments of the under-developed DMSXL mice with TG for the improvement of postnatal survival, increase of growth and restoring of neuromotor activities in CDM1.

The inhibitors of GSK3 also correct the GSK3β-CUGBP1 pathway in human muscle cells from the patients with CDM1 [49,50]. For instance, BIO normalized the downstream myogenic targets downstream of CUGBP1, a transcription factor LEF1; two proteins, associated with cell differentiation, DCX and RBM45 and an extracellular matrix protein, collagen 4A (COL4A; Table 3, ref. [49]).

TG also corrected GSK3β in human CDM1 muscle cells [50]. It seems that this treatment also corrected the CUGBP1 activity because one of the downstream targets of CUGBP1 in myogenesis, RBM45, was corrected in CDM1 muscle cells, treated with TG [50]. The same study showed that the TG treatments improved delayed differentiation of myoblasts, derived from skeletal muscle biopsies of patients with CDM1. As in *HSA^LR^* mice, TG treatments significantly reduced the amounts of the mutant *DMPK* mRNA in human myoblasts from patients with classic DM1 and CDM1 [50]. This reduction of the human mutant *DMPK* mRNA in CDM1 myoblasts was associated with the reduction of CUG-containing RNA foci and with the improvement of splicing activity. Thus, TG or other inhibitors of GSK3 might be beneficial for the reduction of the muscle and cognitive dysfunctions in DM1 and CDM1. The inhibitors of GSK3 might also increase survival in CDM1 and might have a positive effect on the improvement of development.

## 5. Conclusions: What Is Next for Development of CUGBP1-GSK3-Based Therapy in CDM1 and DM1?

Current knowledge, describing the role of CUGBP1 protein in DM1 and CDM1 pathogenesis, suggests that CUGBP1 activity might be corrected in DM1 with the inhibitors of GSK3 kinase. It appears that inhibitor of GSK3, BIO might also correct protein levels of CUGBP1 in DM1 mouse model [49]. It remains to study whether the inhibitors of other kinases, affected in DM1, might also have a positive effect on the correction of GSK3β in DM1. For instance, it is possible, that the modulators of kinases, associated with the control of GSK3β activity in DM1, might restore GSK3β via indirect mechanisms. It has been shown, that the mutant CUG repeats might also deregulate other kinases, besides GSK3β. For instance, CUG repeats elevate PKR kinase (Figure 3, refs. [37,53]). Searches for small molecules, beneficial for DM1 and CDM1, also identified several inhibitors of kinases [95].

There are other signaling pathways, affected in DM1 [98,99,100]. It is possible that the inhibitors of GSK3, such as TG, in addition to GSK3β might indirectly target other signaling pathways, improving DM1 pathology.

Remaining critical questions, related to the reduction of the mutant RNA by the inhibitor of GSK3, TG, include the mechanisms by which TG reduces the toxic RNA in a mouse model of DM1, *HSA^LR^* mice, and in human myoblasts from pediatric CDM1 and adult DM1.

The ability of TG to reduce toxic RNA in DM1 mouse models and DM1/CDM1 human myoblasts shows that the inhibitors of GSK3 may affect not only early toxic events downstream of the mutant CUG repeats, but also target the main cause of DM1 pathogenesis: the mutant CUG-containing RNA. This finding shows that TG may match or compliment other putative treatments for DM1 and CDM1 such as the anti-sense oligonucleotides that cause degradation of the mutant *DMPK* mRNA (rev in 3). However, because of its small size, TG might easily penetrate all tissues, affected in DM1, including CNS. Positive effects of TG in DM1 and DMSXL mouse models suggest that TG might be beneficial in patients with adult, classic DM1 as well as in other clinical forms of DM1, including pediatric and juvenile CDM1. Future clinical studies using large cohort of patients with DM1/CDM1 and thorough analysis of the efficacy and side effects of the inhibitors of GSK3 will show their potential for DM1 and CDM1 therapy.

## Figures and Tables

**Figure 1:**
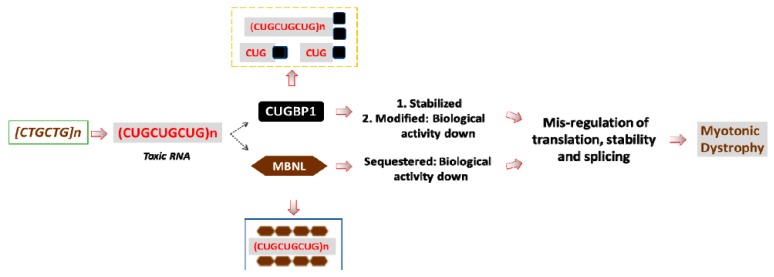
A model for the RNA-based pathogenesis of DM1. The mutant *DMPK* gene with expanded CTG repeats produces the mutant *DMPK* mRNA, containing long CUG repeats. The mutant *DMPK* transcripts have increased stability and, as the result, accumulate within DM1 cells. The mutant RNA has a negative effect on several RNA-binding proteins, including MBNL1 and CUGBP1 (shown by dashed arrows). MBNL1 binds to the stem of the hairpin structures, formed by dsCUG repeats, whereas CUGBP1 binds to the base of the CUG hairpins. CUGBP1 also binds to the mutant CUG repeats in soluble form. As the result, MBNL1 activity is reduced in DM1; whereas the protein levels of MBNL1 remain normal. CUGBP1 protein levels are increased in DM1 due to increased protein stability; however, despite the increased levels of CUGBP1, a portion of the active CUGBP1 is converted into a protein with repressor activity. These changes of MBNL1 and CUGBP1 lead to the alterations of multiple mRNA targets in DM1 patients’ tissues that are normally controlled by these proteins. Other RNA-binding proteins, affected by the mutant CUG repeats, are not shown.

**Figure 2:**
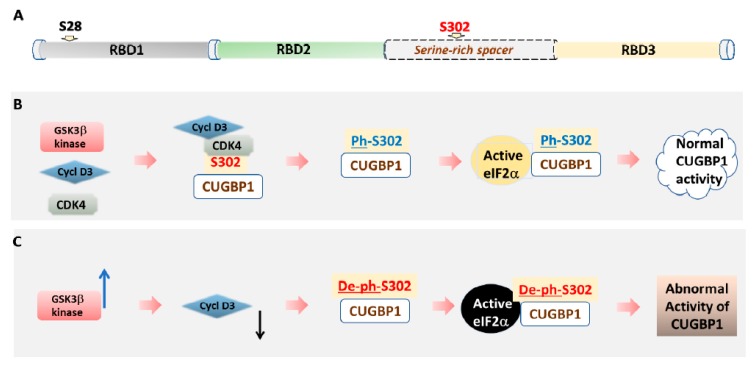
A model of the regulation of CUGBP1 activity by GSK3β-dependent phosphorylation. (**A**) A diagram showing a domain structure of CUGBP1. RNA-binding activity of CUGBP1 depends on the presence of three RNA-binding domains (RBDs). Positions of Ser-28 and Ser-302 are shown. (**B**) In normal cells, cyclin D3-dependent kinase 4 phosphorylates CUGBP1 at Ser-302. Phospho-Ser-302-CUGBP1 forms a complex with active eIF2α. This complex promotes translation on polysomes. (**C**) In DM1 cells, abnormally elevated GSK3β kinase (shown by blue arrow) phosphorylates cyclin D3. As the result, phosphorylated cyclin D3 (shown by black arrow) is subjected to degradation. A reduction of protein levels of cyclin D3 prevents activation of CDK4 and, as the result, CUGBP1 is not phosphorylated at Ser-302. Unphosphorylated at Ser-302 CUGBP1 cannot bind to active eIF2α. Instead, it binds to inactive eIF2α. In-active complexes of eIF2α and un-phospho-Ser-302-CUGBP1 cannot perform normal function in translation, bringing mRNAs to stress granules.

**Figure 3:**
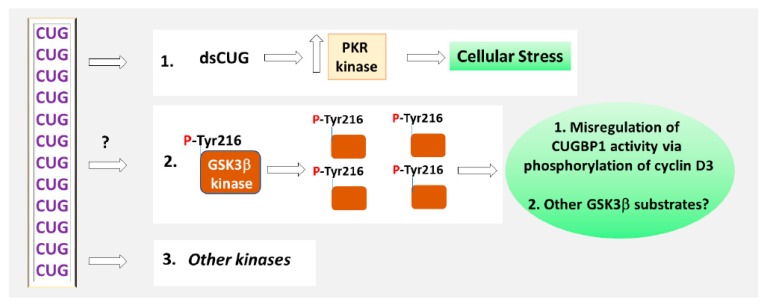
Disruption of the signaling pathways in DM1. The mutant CUG repeats increase PKR kinase, which causes cellular stress in DM1. The toxic CUG-containing RNA increases phosphorylation of GSK3β at Tyr-216. As the result, GSK3β stability is increased in DM1. The elevation of active GSK3β in DM1 cells causes misregulation of CUGBP1 activity via phosphorylation and degradation of one of the substrates of GSK3β, cyclin D3. The alterations of other substrates of GSK3β in DM1 remain to be studied. The mechanism by which the mutant CUG repeats increase phosphorylation of GSK3β at Tyr-216 is unknown. Other kinases might be misregulated by the mutant CUG repeats in DM1 directly and via indirect pathways.

**Table 1:** Inhibitors of GSK3β with a positive effect in the pre-clinical studies of *HSA^LR^* mice.

GSK3 Inhibitor	Effect on the *HSA^LR^* Phenotype	Molecular Effects
Lithium [31]	◾ Reduction of myopathy in adult mice ◾ Reduction of myotonia in adult mice ◾ Reduction of the grip weakness in adult mice	◾ Normalization of GSK3β ◾ Normalization of cyclin D3 ◾ Normalization of CUGBP1 activity
TDZD-8 [31]	◾ Reduction of myotonia in adult mice ◾ Reduction of the grip weakness in adult mice ◾ Activation of myogenic satellite cells in adult muscle	◾ Normalization of GSK3β ◾ Normalization of cyclin D3 ◾ Increase of PAX-7
BIO [49]	◾ Reduction of myopathy in adult mice ◾ Reduction of the grip weakness in adult mice ◾ Prevention of muscle pathology (myopathy and the grip weakness) in adult mice after short treatment at a young age (6 weeks)	◾ Normalization of GSKβ ◾ Normalization of cyclin D3 ◾ Normalization of CUGBP1 levels ◾ Normalization of CUGBP1 activity ◾ Increase of PAX-7 ◾ Normalization of myogenic downstream targets of CUGBP1: RBM45, LEF1, DCX and Col 4A
Tideglusib [50]	◾ Reduction of myopathy in adult mice ◾ Reduction of the grip weakness in adult mice	◾ Reduction of the mutant CUG repeats ◾ Reduction of CUG foci ◾ Normalization of GSK3β ◾ Normalization of myogenic downstream targets of CUGBP1, DCX and RMB45 ◾ Normalization of splicing (*Serca*1, *Cypher*)

**Table 2:** Inhibitors of GSK3 with a positive effect on the phenotype of DMSXL mice, that contain the human *DMPK* gene with more than 1000 CTG repeats in the preclinical studies.

GSK3 Inhibitor	Effect on the Phenotype of DMSXL Mice	Molecular Effects
Tideglusib [50]	◾ Improvement of muscle histopathology in adult mice ◾ Increase of survival after prenatal or postnatal treatment ◾ Increase of postnatal growth after prenatal treatment ◾ Increase of the grip strength after prenatal treatment ◾ Reduction of anxiety after prenatal treatment	◾ Normalization of GSK3β in skeletal muscle and in brain ◾ Normalization of cyclin D3 in skeletal muscle ◾ Increase of Pax-7 ◾ Restoring CUGBP1 activity in brain ◾ Normalization of CUGBP1 downstream targets in brain, *Rbm45*, *MBNL3*, *Smn1* and *Fgf-2*

**Table 3:** Inhibitors of GSK3 with positive effect in human CDM1 and DM1 myoblasts.

GSK3 Inhibitor	Effects on Human CDM1 and DM1 Myoblasts
BIO [49]	◾ Normalization of CUGBP1 myogenic downstream targets (LEF1, DCX, RBM45 and Col4A)
Tideglusib [50]	◾ Improvement of differentiation ◾ Reduction of the mutant *DMPK* mRNA in CDM1 and DM1 myoblasts ◾ Reduction of CUG foci ◾ Normalization of GSK3β ◾ Normalization of myogenic downstream target of CUGBP1, RBM45 ◾ Improvement of splicing of MBNL1 downstream target, *BIN1*

## Abbreviations

AKT	Serine/threonine AKT or Protein kinase B
Ank-2	Ankyrin 2
ARE	AU-rich element
BIN1	Bridging Integrator 1 gene
BIO	6-bromoindirubin-3-oxime
Capzb	F actin capping proteinβ subunit
CDK	Cyclin dependent kinase
CDM	Congenital Myotonic Dystrophy
C/EBPβ	CCAAT-enhancer-binding proteinβ
c-fos	Proto-oncogene
CNS	Central Nervous System
Col	Collagen
CUGBP1/CELF1	CUGBP1 elav-like factor 1
Cx43	Connexin 43
Cypher	Striated Z-line protein
DCX	Doublecortin protein
DM1	Myotonic Dystrophy type 1
DDX	DEAD-box protein
DMPK	Myotonin protein kinase
DMSXL	Mutant mice expressing human *DMPK* gene with more than 1000 CTG repeats
dsCUG	double-stranded CUG RNA
eIF2	Eukaryotic initiation factor 2
FISH	Fluorescence in situ hybridization
FGF-2	Basic fibroblast growth factor 2
Fxr1	Fragile X mental retardation-related protein 1
Fyn	Protein-tyrosine kinase
GRE	GU-rich element
GSK3β	Glycogen synthase kinase 3β
hnRNPs	Heterogeneous nuclear ribonucleoproteins
HO-1	Heme oxygenase 1
HSA^LR^	mutant mice expressing ~250 CTG repeats driven by human skeletal actin promoter
HuR	RNA-binding protein human antigen R
LEF1	Lymphoid enhancer-binding factor 1
MBNL	Muscleblind-like protein
MEF2	Myocyte enhancer factor 2
MYC	Proto-oncogene
MyoD	Myoblast determination protein
PARN	Poly (A)-specific ribonuclease
PAX-7	Paired box protein Pax-7
PB	Processing bodies
PKC	Protein kinase C
PKR	Protein kinase R
PYK2	Protein-tyrosine kinase 2
Rb	Retinoblastoma protein
RBM45	RNA binding motif 45 protein
RyR1	Ryanodine receptor 1
SERCA1	Skeletal muscle sarcoplasmic reticulum Ca^2+^ ATPase 1
Smn1	Spinal motor neuron 1 protein
TDZD-8	4-benzyl-2-methyl-1:2,4-thiadiazolidine-3,5-dione
TG	Tideglusib
TIA1	RNA-binding protein T-cell intracellular antigen-1
TIAR	T-cell intracellular antigen-related protein
TNF	Tumor necrosis factor
TnT	troponin T
Ub	ubiquitin
UTR	untranslated region
